# Appropriateness of Percutaneous Coronary Interventions in Patients With Stable Coronary Artery Disease in US Department of Veterans Affairs Hospitals From 2013 to 2015

**DOI:** 10.1001/jamanetworkopen.2020.3144

**Published:** 2020-04-21

**Authors:** Paul L. Hess, Vinay Kini, Wenhui Liu, Paola Roldan, Patrick Autruong, Gary K. Grunwald, Colin O’Donnell, Jacob A. Doll, P. Michael Ho, Steven M. Bradley

**Affiliations:** 1 University of Colorado Anschutz Medical Campus, Aurora; 2 Rocky Mountain Regional VA Medical Center, Aurora, Colorado; 3 Oregon Health and Science University, Portland; 4 VA Puget Sound Health Care System, Seattle, Washington; 5 Minneapolis Heart Institute, Minneapolis, Minnesota

## Abstract

**Importance:**

In hospitals outside of the US Department of Veterans Affairs (VA) system, 1 in 10 percutaneous coronary interventions (PCIs) for stable coronary artery disease is considered rarely appropriate by the appropriate use criteria, with variation across hospitals. The appropriateness of PCIs in VA hospitals has not been documented.

**Objective:**

To characterize the appropriateness of PCIs in VA hospitals.

**Design, Setting, and Participants:**

This retrospective cohort study included patients with stable coronary artery disease undergoing elective PCI from November 1, 2013, to October 31, 2015, within the VA Clinical Assessment, Reporting, and Tracking Program, an operational program that includes 59 VA hospitals. Data were analyzed from March 1, 2019, to August 8, 2019.

**Exposures:**

Elective PCI at a VA hospital.

**Main Outcomes and Measures:**

Proportion of PCIs classified as appropriate, may be appropriate, or rarely appropriate; extent of hospital-level variation in rarely appropriate PCIs using criteria issued by cardiovascular professional societies in 2012. The extent of hospital-level variation in rates of rarely appropriate PCI was characterized using hospital proportions and random-effect logistic regression.

**Results:**

Among 2611 patients undergoing elective PCI (mean [SD] age, 66.3 [7.6] years; 2577 [98.7%] men) at 59 hospitals, a total of 778 PCIs (29.8%) were classified as appropriate, 1561 PCIs (59.8%) were classified as may be appropriate, and 272 PCIs (10.4%) were classified as rarely appropriate. Rarely appropriate PCIs were more commonly performed in patients who had low-risk stress test findings (220 patients [89.1%]), who were taking no (100 patients [36.8%]) or 1 (167 patients [61.4%]) antianginal medication, or who had 1 coronary artery stenosis (185 patients [68.0%]). The unadjusted hospital-level rates of rarely appropriate PCIs ranged from 0% to 28.6%, with a median (interquartile range) of 9.7% (6.3%-13.9%). Random-effect models yielded an estimated median (interquartile range) rate of rarely appropriate PCI of 10.4% (8.7%-12.3%).

**Conclusions and Relevance:**

These findings suggest that in VA practice, most PCIs for stable coronary artery disease were classified as appropriate or may be appropriate. However, 1 in 10 PCIs was classified as rarely appropriate, with variation across VA hospitals. Efforts to improve patient selection are needed.

## Key Points

**Question:**

What is the rate of appropriateness of percutaneous coronary interventions (PCIs) in US Department of Veterans Affairs (VA) hospitals?

**Findings:**

In this cohort study of 2611 patients with stable coronary artery disease undergoing elective PCI within the VA Clinical Assessment, Reporting, and Tracking Program, 29.8% of PCIs were classified as appropriate, 59.8% of PCIs were classified as may be appropriate, and 10.4% of PCIs were classified as rarely appropriate. There was variation in the estimated proportion of rarely appropriate PCIs across VA hospitals.

**Meaning:**

These findings suggest that in contemporary VA practice, most PCIs are classified as appropriate or may be appropriate; however, given that 1 in 10 PCIs was classified as rarely appropriate, efforts to improve patient selection are needed.

## Teaser

This cohort study assesses the appropriateness of percutaneous coronary interventions (PCIs) among patients with stable coronary artery disease in the US Department of Veterans Affairs (VA) health system and analyzes the interhospital variation in rates of inappropriate PCIs.

## Introduction

Approximately 200 000 elective percutaneous coronary interventions (PCIs) are performed annually in the US.^1^ In the setting of stable coronary disease, PCI offers the potential for anginal symptom relief but does not improve survival or reduce the risk of myocardial infarction.^2,3^ Potential periprocedural complications include bleeding, acute kidney injury, vascular access complications requiring treatment, stroke, tamponade, arrhythmia, and death.^4^ Moreover, attributable annual costs exceed $3 billion.^5^ Appropriate patient selection is therefore critical.

The PCI appropriate use criteria were developed by cardiovascular professional societies as a means of optimizing patient care.^6^ They synthesize clinical trial data, practice guidelines, and expert opinion to categorize clinical scenarios as appropriate care, may be appropriate care, and rarely appropriate care.^7,8^ A 2011 study reported that while most acute cases were appropriate, 1 in 10 nonacute PCIs was rarely appropriate, with variation in rates of rarely appropriate PCIs across hospitals.^9^ However, to our knowledge, PCI appropriateness rates are unknown in the US Department of Veterans Affairs (VA) health care system, the largest integrated health care system in the US. Accordingly, we sought to assess overall rates of PCI appropriateness, to understand the most common clinical scenarios in which inappropriate PCIs occur, and to characterize hospital-level variation in rarely appropriate PCIs for stable coronary disease in the VA.

## Methods

This cohort study was approved by the Colorado Multiple Institution Review Board with a waiver of informed consent given minimal risk exposure. This study is reported following the Strengthening the Reporting of Observational Studies in Epidemiology (STROBE) reporting guideline.

### Data Sources and Study Population

The VA Clinical Assessment, Reporting, and Tracking system is a national quality improvement program centered on cardiovascular procedures performed in all cardiac catheterization laboratories across the VA health care system and has been described previously.^10^ A cornerstone of the program is a clinical software application integrated into the VA electronic health record that captures clinical information at the point of care for purposes of quality monitoring and improvement.^10^ Data elements are comparable to those of the National Cardiovascular Data Registry CathPCI, and the data set is routinely validated for accuracy and completeness.^11^

For the current analysis, all patients who underwent elective PCI for stable coronary disease between November 1, 2013, and October 31, 2015, in the VA health system were identified. Patients undergoing catheterization for purposes of acute coronary syndrome, preoperative assessment, valvular heart disease, or cardiomyopathy only or who did not have coronary disease were excluded. Patient race/ethnicity was recorded in the VA Corporate Data Warehouse, which contains the most recent race/ethnicity entered in the Veterans Information Systems and Technology Architecture system. In the event multiple entries were found, priority was given to whichever race/ethnicity was self-reported or reported by a proxy. Trained personnel then abstracted data from individual patient records on patient symptoms and stress testing results and recorded their findings in real-time in an electronic database. To understand interrater reliability, 330 records were randomly selected for abstraction by several reviewers and yielded a Cohen κ of 0.95 (95% CI, 0.92-0.97). Two of us who are certified in cardiovascular disease by the American Board of Internal Medicine (P.L.H. and V.K.) then adjudicated each stress test according to the level of risk using appropriate use criteria issued in 2012.^7^ These data were then linked to cardiac catheterization report documentation as well as clinical pharmacy data.

### Appropriate Use Criteria Element Definitions

Appropriate use criteria issued in 2012 by the American College of Cardiology Foundation Appropriate Use Criteria Task Force, Society for Cardiovascular Angiography and Interventions, Society of Thoracic Surgeons, American Association for Thoracic Surgery, American Heart Association, American Society of Nuclear Cardiology, and the Society of Cardiovascular Computed Tomography served as the source of element definitions in the primary analysis.^7^ Accordingly, maximal medical therapy was defined as using 2 or more classes of antianginal drugs, including β-blockers, calcium channel blockers, nitrates, and ranolazine. Significant stenoses were defined as 70% or more lesions in the left anterior descending artery, left circumflex artery, or right coronary artery or 50% or more lesions in the left main artery. Fractional flow reserve data were used when available (6% of records); significant stenoses were defined as 0.80 or less. Given the nature of real-world clinical documentation, anginal symptoms were not reported according to the Canadian Cardiovascular Society classification system in most records. Therefore, patient symptoms (eg, chest pain or dyspnea) were dichotomized as either present or absent. In a sensitivity analysis, appropriate use criteria issued in 2017 by the American College of Cardiology Appropriate Use Criteria Task Force, American Association for Thoracic Surgery, American Heart Association, American Society of Echocardiography, American Society of Nuclear Cardiology, Society for Cardiovascular Angiography and Interventions, Society of Cardiovascular Computed Tomography, and Society of Thoracic Surgeons served as the source of element definitions.^8^

### Statistical Analysis

The proportion of PCIs classified as appropriate, rarely appropriate, or may be appropriate was evaluated. Demographic and clinical characteristics of patients, as well as key variables in the assessment of appropriateness, including the presence or absence of symptoms, level of risk indicated by noninvasive stress testing, and number of antianginal medications, were compared by groups of appropriateness. Categorical data were assessed with the χ^2^ test, and continuous data were assessed using the Kruskal-Wallis test.

To characterize hospital-level variation in inappropriate PCIs, only sites with 10 or more PCIs during the study were included. Hospital proportions of rarely appropriate PCIs were summarized with medians and interquartile ranges (IQRs). Given that raw hospital proportions can vary owing to small and variable hospital sizes and volumes, we fitted a logistic regression model with a hospital normal random intercept. Medians and IQRs were calculated from the estimated intercept and random effect SD, transformed to the probability scale. Since factors used to assess appropriateness may correlate with several patient factors, patient factors were not included in this model. Analyses were performed using SAS statistical software version 9.4 (SAS Institute). *P* values were 1-sided, and statistical significance was set at .05.

## Results

Among 2611 patients who underwent elective PCIs for stable coronary disease (mean [SD] age, 66.3 [7.6] years; 2577 [98.7%] men; 2238 white patients [85.7%]), 1151 patients (44.1%) had a history of PCI, 1324 patients (50.7%) had diabetes, 2372 patients (90.8%) had hypertension, 2419 patients (92.6%) had dyslipidemia, and 1800 patients (68.9%) had a history of tobacco use. A total of 778 PCIs (29.8%) were classified as appropriate, 272 PCIs (10.4%) were classified as rarely appropriate, and 1561 PCIs (59.8%) were classified as may be appropriate. While most characteristics were not clinically significant across groups of appropriateness, patients receiving appropriate PCIs more commonly had history of myocardial infarction (315 patients [40.5%] with appropriate PCIs; 78 patients [28.7%] with rarely appropriate PCIs; 554 patients [35.5%] with may be appropriate PCIs; *P* < .001), PCI (376 patients [48.3%] with appropriate PCIs; 103 patients [37.9%] with rarely appropriate PCIs; 671 patients [43.0%] with may be appropriate PCIs; *P* = .005), or coronary artery bypass grafting (245 patients [31.5%] with appropriate PCIs; 32 patients [11.8%] with rarely appropriate PCIs; 280 patients [17.9%] with may be appropriate PCIs; *P* < .001) (Table 1). In unadjusted analyses, most rarely appropriate PCIs occurred among patients with low-risk stress test findings (220 patients [89.1%]), using no (100 patients [36.8%]) or 1 (167 patients [61.4%]) antianginal medication, or with 1 coronary artery stenosis on coronary angiography (185 patients [68.0%]) (Table 2). In a sensitivity analysis using appropriate use criteria issued in 2017,^8^ 719 PCIs (39.5%) were classified as appropriate, 71 PCIs (3.9%) were classified as rarely appropriate, and 1033 PCIs (56.7%) were classified as may be appropriate.

**Table 1.** Patient Characteristics

Characteristic	No. (%)				*P* value
	Total (N = 2611)	Appropriate (n = 778)	Rarely appropriate (n = 272)	May be appropriate (n = 1561)	
Age, mean (SD), y	66.3 (7.6)	66.9 (7.8)	65.2 (7.5)	66.2 (7.6)	.002
Men	2577 (98.7)	762 (97.9)	271 (99.6)	1544 (98.9)	.05
Race/ethnicity					
White	2238 (85.7)	680 (87.4)	230 (84.6)	1328 (85.1)	.58
African-American	309 (11.8)	81 (10.4)	35 (12.9)	193 (12.4)	
Other	64 (2.5)	17 (2.1)	7 (2.5)	40 (2.6)	
Comorbidity					
Prior MI	947 (36.3)	315 (40.5)	78 (28.7)	554 (35.5)	.001
Prior PCI	1151 (44.1)	376 (48.3)	103 (37.9)	671 (43.0)	.005
Prior CABG	557 (21.3)	245 (31.5)	32 (11.8)	280 (17.9)	<.001
Heart failure	541 (20.7)	197 (25.3)	33 (12.1)	311 (19.9)	<.001
Diabetes	1324 (50.7)	431 (55.4)	135 (49.6)	758 (48.6)	.007
Hypertension	2372 (90.8)	726 (93.3)	237 (87.1)	1409 (90.3)	.004
Dyslipidemia	2419 (92.6)	730 (93.8)	251 (92.3)	1438 (92.1)	.32
Hemodialysis	60 (2.3)	9 (1.2)	11 (4.0)	40 (2.6)	.01
Cerebrovascular disease	463 (17.7)	161 (20.7)	42 (15.4)	260 (16.7)	.03
Peripheral arterial disease	517 (19.8)	175 (22.5)	59 (21.7)	283 (18.1)	.03
Chronic lung disease	720 (27.6)	197 (25.3)	67 (24.6)	456 (29.2)	.07
Tobacco use	1800 (68.9)	534 (68.6)	183 (67.3)	1083 (69.4)	.77

Abbreviations: CABG, coronary artery bypass grafting; MI, myocardial infarction; PCI, percutaneous coronary intervention.

**Table 2.** Key Appropriate Use Variables

Variable	No. (%)				*P* value
	Total (N = 2611)	Appropriate (n = 778)	Rarely appropriate (n = 272)	May be appropriate (n = 1561)	
Chest pain or dyspnea	2515 (96.3)	776 (99.7)	216 (79.4)	1523 (97.6)	<.001
Noninvasive evaluation results					
Low risk	378 (18.1)	15 (1.9)	220 (89.1)	143 (13.5)	<.001
Intermediate risk	1049 (50.3)	239 (30.7)	26 (10.5)	784 (73.8)	
High risk	639 (30.6)	524 (67.4)	0	115 (10.8)	
None performed	526 (20.9)	0	25 (9.2)	501 (32.1)	
Antianginals, No.					
0	621 (23.8)	96 (12.3)	100 (36.8)	425 (27.2)	<.001
1	1305 (50.0)	260 (33.4)	167 (61.4)	878 (56.2)	
2	645 (24.7)	400 (51.4)	5 (1.8)	240 (15.4)	
3	40 (1.5)	22 (2.8)	0	18 (1.2)	
Coronary artery stenoses					
1	1560 (59.7)	404 (51.9)	185 (68.0)	971 (62.2)	<.001
2	702 (26.9)	215 (27.6)	76 (27.9)	411 (26.3)	
3 or left main	332 (12.7)	154 (19.8)	11 (4.0)	167 (10.7)	

A total of 66 VA hospitals were analyzed, and 7 hospitals were excluded because fewer than 10 PCIs were performed there. Among the remaining 59 hospitals, we found hospital-level variation in the proportion of rarely appropriate procedures according to 2012 criteria.^7^ The unadjusted proportion of inappropriate PCIs ranged from 0% to 28.6% with a median (IQR) of 9.7% (6.3%-13.9%) (Figure). Using the SD from the model including a random hospital intercept, the estimated median (IQR) proportion of rarely appropriated PCIs per site was 10.4% (8.7%-12.3%).

**Figure.**
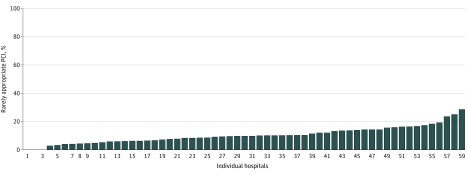
Variation in the Proportion of Rarely Appropriate Percutaneous Coronary Interventions (PCIs) Across Hospitals The unadjusted proportion of rarely appropriate PCIs ranged from 0% to 28.6% with a median (interquartile range) of 9.7% (6.3%-13.9%).

## Discussion

This cohort study examining the appropriateness of elective PCI for stable coronary disease in the VA health care system, the largest integrated health care system in the US, yielded 3 main findings. First, most procedures were classified as appropriate or may be appropriate. Second, 1 in 10 procedures was classified as rarely appropriate and most commonly occurred in the context of patients with low-risk stress testing results, suboptimal antianginal medication use, or a single significant coronary artery stenosis. Third, there was variation in the rate of rarely appropriate procedures across hospitals.

Data from the American College of Cardiology’s National Cardiovascular Data Registry CathPCI indicates that most PCIs performed in non-VA hospitals in the US from 2009 to 2010 were appropriate. However, 11.6% of elective PCIs were classified as rarely appropriate, with variation across hospitals (median [IQR] rate, 10.8% [6.0%-16.7%]). In our study, most rarely appropriate PCIs occurred in the context of low-risk findings on stress testing (89.1%), insufficient (≤1 medication) antianginal therapy (95.8%), or 1 significant coronary artery stenosis (68.0%).^9^ Studies based on regional quality improvement programs have had comparable findings.^12,13^ Our analysis suggests that VA practice patterns are comparable to those observed in non-VA hospitals but with less variation across hospitals, which may indicate that care standards are applied more consistently across the VA health care system, may reflect variability in hospital sample sizes, or may be associated with both.

There may be several reasons underlying inappropriate PCIs. Rarely appropriate PCIs may stem from a desire for increased physician or hospital income in a fee-for-service health care system. This theory has gained traction in the medical literature and lay press.^14^ However, VA employees are salaried and thus do not have an underlying profit motive. That some level of rarely appropriate use remains in the absence of a physician profit motive suggests that the distribution of classifications is not a simple manifestation of a desire for increased physician reimbursement. Rather, other factors appear operative in the VA and perhaps elsewhere. In the VA, for example, hospital-level reimbursement is tied in part to the number of patients seen rather than volume of patients in the catheterization laboratory, and this may indirectly incentivize maintaining or increasing procedural volume. Clinician-related factors may include a lack of awareness, familiarity, or agreement with the appropriate use criteria.^15,16^ Patients’ overestimation of the potential benefit of the procedure^17^ and, in turn, desire for it^18^ may also play a role. Collectively, these issues may lead to continued inertia in practice change. Alternatively, documentation justifying PCI may be insufficient. Additional study of institutional-, clinician-, and patient-level factors is needed.

Variation in hospital-level performance in rarely appropriate PCIs was present. Additional study of underlying patient factors, such as medication adverse effects or intolerances, may prove helpful. In addition, quality improvement initiatives may be used across a number of VA catheterization laboratories. Efforts to improve PCI appropriateness may include hospital or cardiac catheterization laboratory leadership that prioritizes appropriate use criteria performance, oversight of PCI appropriateness accompanied by internal review of processes designed to achieve optimal performance,^19^ check lists, or decision support tools.^20^ Hospital-based systems have the potential to improve the quality of care by enhancing the likelihood of proper patient selection, which may in turn maximize procedural benefit while simultaneously minimizing procedural complications and costs.

### Strengths and Limitations

There are several strengths of this analysis, including a large and nationally representative study sample and a high-quality data set. Our study included abstracted symptoms and adjudicated stress testing results, the absence of which has hampered prior analyses.^9,12,21^

Our study also has several limitations. First, patient symptoms were abstracted from the medical record and thus dependent on clinicians’ assessment. Second, symptoms of chest pain or shortness of breath were dichotomized as present or absent rather than classified according to Canadian Cardiovascular Society angina class. This may have led to a smaller proportion of procedures classified as rarely appropriate. Third, low use of antianginal medications among patients receiving a PCI classified as rarely appropriate may reflect medication adverse effects or nonadherence. Fourth, a 2019 study^22^ reported that PCI appropriateness indicators may be “gamed” by upcoding patient symptoms, thereby making unnecessary procedures seem necessary. Future efforts at understanding PCI appropriateness may incorporate patient-reported health status, including symptom burden, functional status, and health-related quality of life,^23^ to reduce the potential for misclassification or “gaming.” Fifth, limitations of the appropriate use criteria described previously include a lack of granularity regarding how to classify PCIs of chronic total occlusions.^16^ Sixth, after the study period, updated appropriate use criteria, published in 2017, reclassified a number of procedures previously categorized as rarely appropriate into the may be appropriate category.^8^ However, we chose to use the 2012 criteria for the primary analysis because it was operative during the study. In addition, it allowed for direct comparison with prior appropriate use criteria studies, most of which also used 2012 criteria. It is important to note that several important studies have been performed since the 2017 criteria were issued, including the Objective Randomized Blinded Investigation with optimal medical Therapy of Angioplasty in stable angina (ORBITA) trial^24^ and International Study of Comparative Health Effectiveness With Medical And Invasive Approaches (ISCHEMIA) trial.^25^ The ORBITA trial demonstrated that PCI did not increase exercise time more than medical therapy among patients with medically treated angina and significant coronary artery stenosis.^24^ The ISCHEMIA trial showed that revascularization by PCI did not confer a reduction in a composite end point of cardiovascular death, myocardial infarction, hospitalization for unstable angina, and heart failure or resuscitated cardiac arrest compared with medical therapy among patients with stable ischemia heart disease.^25^ These studies may or may not influence future iterations of appropriateness criteria. Seventh, the objective of our study was to characterize PCI appropriateness; therefore, potential PCI underuse^26^ was not captured. Eighth, the study was observational in nature, leaving open the possibility of unmeasured or residual confounding. However, given the intent to assess PCI appropriateness in routine clinical practice, an observational study design was the only feasible approach.

## Conclusions

The findings of this cohort study indicate that most PCIs performed in the VA were appropriate or may be appropriate. However, 1 in 10 PCIs was rarely appropriate, and there was a small amount of variation in the rates of rarely appropriate procedures across hospitals. Efforts to improve patient selection and, in turn, patient outcomes are needed.
